# Food for thought: The role of life cycle thinking in sustainable food system transitions

**DOI:** 10.1038/s44319-026-00853-x

**Published:** 2026-07-01

**Authors:** Nathan Pelletier, Sofia Bahmutsky, Nicole Bamber, Jared Brown, Lauren McNeil, Nicole Sibanda, Shaiyan Siddique, Amandeep Singh, Ian Turner

**Affiliations:** 1 University of British Columbia, Biology, Kelowna, BC Canada; 2 University of British Columbia, IGS Sustainability, Kelowna, BC Canada

**Keywords:** Biotechnology & Synthetic Biology, Economics, Law & Politics, Methods & Resources

## Abstract

Multi-criteria sustainability analyses, based on life cycle thinking and assessment, are critical to help us navigate sustainable transitions in food and agriculture. This includes current industrial agriculture as well as new technologies such as vertical farming, cellular agriculture and macronutrient synthesis that may well transform the future of food.

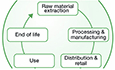

Humanity’s collective activities now exceed Earth’s carrying capacity in several critical areas (Morshed et al, 2024). As the human population, its environmental impact, and socioeconomic inequalities continue to grow, sustainable development (SD) has become a central goal for governments, industries, and local communities. However, SD remains difficult to achieve in practice as it often requires solving complex issues defined by multiple competing objectives spanning environmental stewardship, social justice, and economic prosperity. The 17 UN Sustainable Development Goals (SDGs) constitute the closest humanity has yet come to a consensus vision for a sustainable future, but how to achieve these goals remains heavily debated.

Food production systems are central to many of our most pressing environmental sustainability challenges, including being the principal cause of land use change, biodiversity loss, freshwater use, altered biogeochemical flows of nitrogen and phosphorus, and a major source of greenhouse gas (GHG) emissions (Kemarau et al, 2025). They are similarly directly or indirectly linked to achieving almost all of the SDGs. While obviously critical for food security, and directly supporting the SDGs of “zero hunger” (SDG 2) and “good health and well-being” (SDG 3), strategies to address these SDGs may also positively or negatively impact others such as “clean water and sanitation” (SDG 6), “life below water” (SDG 14), and “life on land” (SDG 15). Most “solutions” will engender trade-offs, where progress towards some goals may hinder others. Moreover, priorities with respect to specific SDGs will differ between groups, industries, and societies, and must be navigated to address food-system sustainability challenges.

Food production systems are central to many of our most pressing environmental sustainability challenges…

## Life cycle thinking and assessment

Outside of limited examples of pre-industrial agriculture, food production systems are invariably underpinned by complex, often globe-spanning supply chains that generate a wide range of environmental, social, and economic benefits and impacts. Solutions that genuinely support long-term sustainability goals are hence neither simple nor obvious. Understanding their ramifications requires tools that employ a systems-based, multi-criteria analytical approach. Life Cycle Thinking (LCT) analyses how activities along food and other supply chains are interconnected, and how decisions in one part of the system can influence outcomes elsewhere, including potential synergies and trade-offs across a variety of sustainability aspects (Hauschild et al, 2018). For example, switching to lighter packaging material to reduce fuel consumption during transportation may generate higher emissions during manufacturing or decrease recyclability—a phenomenon known as “burden shifting”.

Life Cycle Thinking […] analyses how activities along food and other supply chains are interconnected, and how decisions in one part of the system can influence outcomes elsewhere…

While LCT provides the conceptual lens, life cycle assessment (LCA) is the most widely utilized method for operationalizing LCT to support evidence-based policy and management decisions (Hauschild et al, 2018). Environmental LCA is used to quantify material and energy inputs  and outputs—pollution, GHG and other emissions—along the supply chain of a product or service in order to estimate the net environmental impacts on a multi-criteria basis: climate change, water use, energy and land use, and so on.

**Figure. 1:**
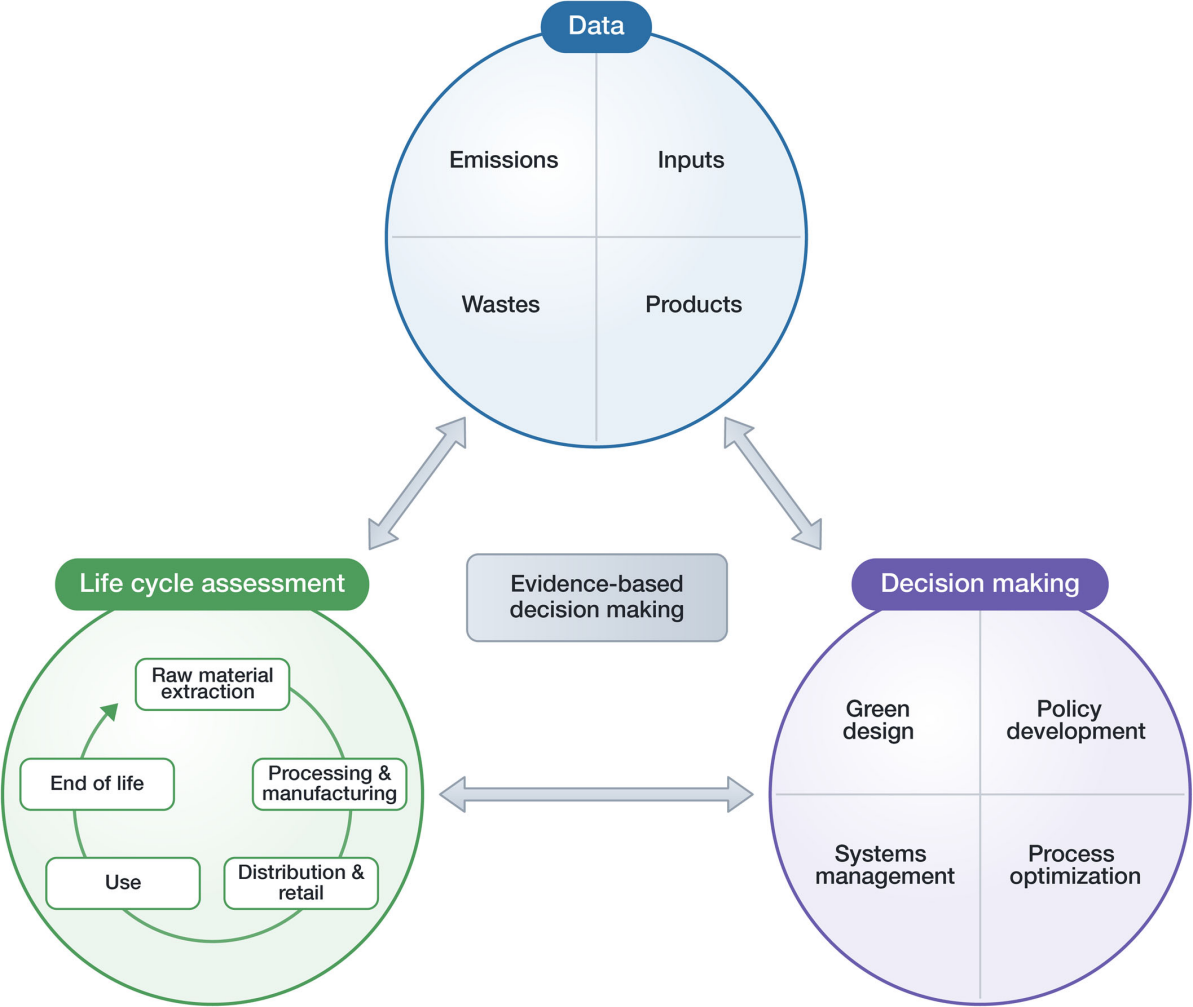
The relationships among life cycle assessment, data, and evidence-based decision-making.

Application of this method is regulated by the ISO 14044 standard, which describes LCA as comprising four distinct, although often iterative, methodological stages: Goal and Scope definition, Life Cycle Inventory (LCI), Life Cycle Impact Assessment (LCIA) and Life Cycle Interpretation (Fig. 1). The Goal and Scope phase defines the target system, along with the research questions, audience, applications and limitations. This includes all system boundaries, impacts to be assessed, assumptions, data sources, and so on, effectively creating a transparent and reproducible map of the planned analysis. The data required to operationalize the study are collected during the Life Cycle Inventory stage. The final output of this stage is a detailed compilation of all material and energy resource flows and emissions for each stage of the product/service life cycle that cross the boundary between the “ecosphere” – the supporting biophysical environment – and the human “technosphere”. The Impact Assessment classifies these flows in terms of relevant environmental impact categories and characterizes the impact of each flow relative to a reference species (category indicator). This enables aggregating impact contributions in each category to an overall indicator– for example, the carbon footprint for the life cycle of a product. The Life Cycle Interpretation applies data-quality and consistency checks to ensure alignment with the defined study scope and interprets the results relative to the originally defined goals.

For industry, LCA studies can be used to guide product design, optimize logistics, or compare the environmental performance of a food product or technology alternatives. From a government perspective, LCA can assist in the formulation, implementation, and evaluation of environmental policies (Hauschild et al, 2018). Environmental LCA is, by far, the most well-developed and commonly applied life cycle-based assessment method but it is increasingly complemented by social life cycle assessment and life cycle costing, providing the basis for integrated life cycle sustainability assessment.

## Lessons learned from LCA of contemporary food systems

Environmental LCA has already been widely applied to assess diverse food product supply chains to understand the general magnitude and distribution of sustainability challenges, as well as to evaluate potential solutions. For example, LCA has established that ruminant meat has, by far, the highest impacts of any major terrestrial protein source, with GHG emissions as much as 100 times higher than plant-based proteins (Poore and Nemecek 2018). However, while animal products are generally more resource and emissions intensive, there is considerable differences between them: swine and poultry products are far less impactful than ruminant products. LCA has also taught us to be wary of rules of thumb and to consider context-specific details when comparing food alternatives. For example, dried beans boiled over a gas range for several hours to render them palatable may be comparable in GHG intensity to some animal products, whereas pressure cooking them would add only a small margin to their carbon footprint (Frankowska et al, 2020).

LCA has also taught us to be wary of rules of thumb, and to consider context-specific details when comparing food alternatives.

LCA has also been instrumental in challenging widely held assumptions about food and sustainability, such as the notion that eating locally produced food is necessarily a more sustainable choice because it reduces “food miles”. Unless air freighted, LCA studies have repeatedly demonstrated that transportation often contributes only a small share of GHG emissions and other impacts. Bamber et al, (2025), for example, reported that field crops grown in Canada could be shipped up to an additional 17 times to Europe, before it would be less GHG-intensive to produce them domestically, due to lower production-related impacts in Canada, and the relatively small emission contributions made by transportation via rail and ocean freight.

Another common misconception is that grass-finished beef has a lower carbon footprint than beef from grain-finished animals. Pelletier et al (2010) found that grass-finished beef had considerably higher life-cycle resource use and emissions due to longer finishing times, and higher enteric fermentation and manure emissions. Similarly, while reusable grocery bags are often promoted as more sustainable than plastic bags, the UN Environment Programme found that a cotton bag must be used 50-150 times for it to be less GHG-intensive than a single-use plastic bag.

However, being attentive to what is being measured and compared—and ensuring that we measure and compare the things that really matter—is critical. Single-use plastics like disposable grocery bags are a major source of macro and microplastic pollution – aspects that are not well characterized, if at all, in most LCA studies. Moreover, beyond environmental sustainability concerns, food choices impact a plethora of socioeconomic aspects. Local food, for example, might better support objectives related to domestic employment, food sovereignty, or cultural values. Similarly, grass-finished beef may support better nutritional profiles and animal welfare (Carrillo et al, 2016).

## LCA and the future of food

While LCA has been highly useful for identifying opportunities to improve sustainability outcomes in conventional food and agriculture, it seems increasingly unlikely that such solutions alone will be sufficient to achieve the magnitude of sustainability improvements that are required. This is particularly salient in light of continued population growth, urbanization, the shrinking proportion of farmers and dietary transitions towards more resource-intensive, higher impact diets. Indeed, traditional agriculture encompasses just a small subset of the technologies that have together propelled our species to where we are today and may simply be decreasingly relevant in the future. Instead, profound system transformation will likely be both necessary and inevitable.

Already, traditional agriculture is beginning to give way to new modes of food production. Some of these, such as vertical farming, are comfortingly recognizable based on the familiarity of the foods that are produced (Barbosa et al, 2015). Others, like land-based algae cultivation, bear passing resemblance to traditional, soil-based agriculture, but entail potential changes in the basic macronutrient building blocks of our food systems (Novoveská et al, 2023). Still other food production methods, like cultivation of animal cells and precision fermentation—cellular agriculture—or direct industrial synthesis of macronutrients are unrecognizable from contemporary agriculture (Bergman et al, 2024).

Against this backdrop, concerted use of LCT/LCA will be critical in shaping the development of these technologies that may well redefine humanity’s relationship to food, and the numerous ways in which they may help to resolve or exacerbate sustainability challenges. Important insights are already emerging as researchers begin to apply LCA to evaluate “future food” technologies. For example, high-value, fast-growing plants such as lettuce, spinach, kale, and basil are increasingly grown in indoor vertical farms that can close nutrient cycles, exclude pests, recycle water, and reduce waste. This comes at the expense, however, of higher energy inputs. Vertical farming of lettuce in Arizona, for example, reduced water and land use by more than 90% compared to conventional production, but increased energy consumption 82-fold (Barbosa et al, 2015). Such findings underscore the critical importance of developing future food technologies that can be supported by low impact, renewable energy sources.

… concerted use of LCT/LCA will be critical in shaping the development of technologies that may well redefine humanity’s relationship to food…

Alternative technologies may support new ways of producing food either for direct human consumption or for use as animal feed, which is currently a major driver of resource use and impacts. Such technologies hold considerable promise when the substrates employed are derived from upcycled food waste—a key consideration for reducing landfill gas emissions—or other forms of low-opportunity cost biomass (LOCB) (Smetana et al, 2016). Insects reared on LOCB substrates have long been heralded as a potentially sustainable protein source for direct human consumption with about 30% of the net environmental impacts of chicken. However, while insects are widely consumed globally, cultural norms limit their direct consumption in many cultures. Instead, they might substitute protein ingredients in livestock feeds as insects can have a lower impact than even plant-derived protein feed ingredients, provided they are produced using best available technologies and low-impact feedstocks (Smetana et al, 2016).

Meat production for direct human consumption via cellular agriculture is another development that may considerably lessen environmental impacts by reducing feed production, manure management and, in the case of ruminants, enteric methane emissions. Production of seafood products based on cellular agriculture similarly has great potential to reduce pressures on marine environments and wild fisheries—many of which have been overfished beyond carrying capacity with severe ecological consequences. However, cellular agriculture comes with its own set of burdens, particularly from the resource demands of the culture medium, cell scaffolds, and the energy to operate bioreactors (Orsini et al, 2026). LCAs show consistent reductions in land and water use for cultured meat compared to traditional animal products, up to 96–99% lower than beef. Yet, cellular agriculture can only be considered a more environmentally friendly alternative if resource use and energy-related impacts can be further mitigated as technologies continue to evolve and scale (Orsini et al, 2026).

… cellular agriculture can only be considered a more environmentally friendly alternative if resource use and energy-related impacts can be further mitigated…

In addition to food production for direct human consumption, cellular agriculture technology, such as precision fermentation, can also be used to produce ingredients for animal feed from waste streams, offering the dual benefit of reducing input resource requirements and mitigating emissions from landfills. For example, filamentous fungi as a protein feed was shown to reduce feed-related environmental impacts of farmed rainbow trout, compared to conventional ingredients like soy protein (Bergman et al, 2024). This was true for all impacts except energy use, which was 21% higher (Bergman et al, 2024). Again, this underscores the trade-offs of generally higher energy use associated with these novel technologies compared to traditional methods of food and nutrient production. The clean energy transition will therefore be critical to mitigate these energy-related environmental and resource-depletion impacts.

An even more radical departure from traditional food production is direct chemical synthesis of nutrients from industrially fixed carbon, nitrogen, and other materials. While methods for synthesizing macronutrients have been available for decades in other industrial contexts (Hudlicky et al, 1996), they have yet to be practically implemented in food production. If implemented, such technologies could bypass biological photosynthesis, which is a relatively inefficient process, leading to large reductions in land, water, and agrochemical use, new possibilities for atmospheric CO_2_ capture, and improvements in nutrient-use efficiency. There are also additional knock-on benefits, such as biodiversity improvements that could result from the re-wilding of agricultural land. Realization of these sustainability benefits, however, is contingent on continued technological innovation focused on reducing energy use and scalability (O’Brien et al, 2022).

Yet, while these strategies to potentially revolutionize the future of food and agriculture are exciting, most remain at pre- or early-commercial development levels, hence characterization of their potential sustainability benefits is challenging, and may often be underestimated. For example, a recent LCA study of a new, commercial-scale black soldier fly production facility in the USA found that a 60-fold reduction in environmental impacts was likely achievable via process and technological efficiency improvements (Domínguez Aldama, 2023). Similarly, impacts of single-cell protein production have been shown to decrease from 60 to 96% across all impacts modeled when scaling up from laboratory-scale to industrial-scale production (Martínez-Ibáñez et al, 2025).

## The future of LCA

**Figure. 2:**
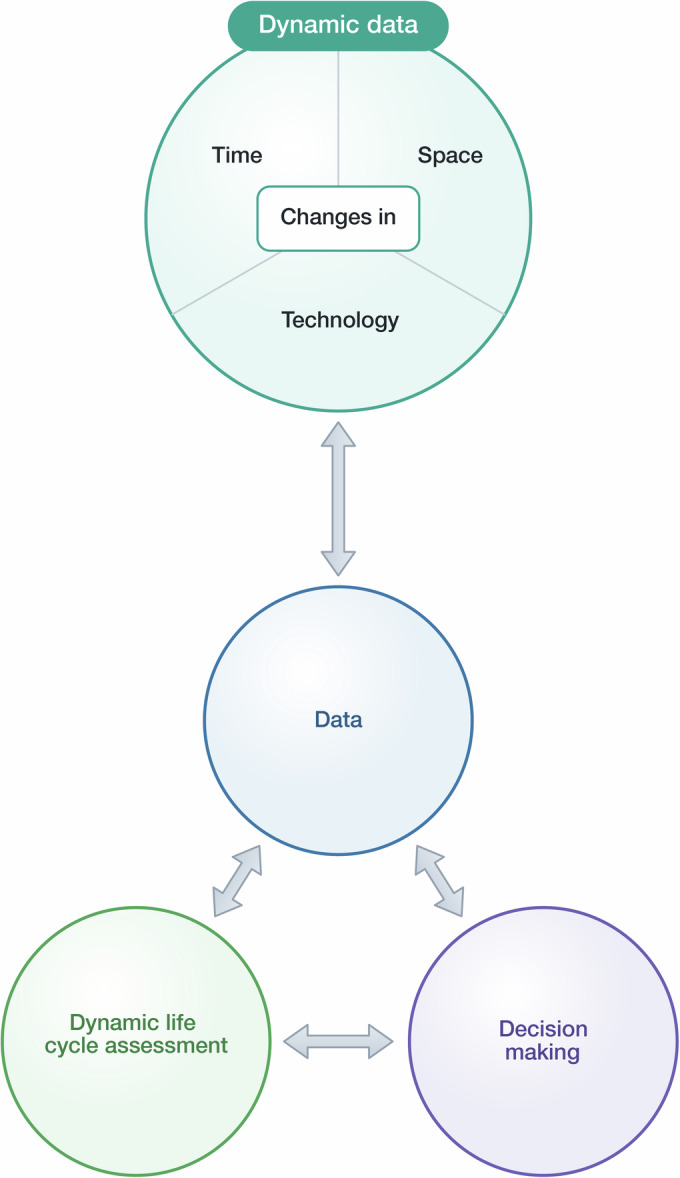
Accounting for changes in time, space, and technology using dynamic LCA for prospective sustainability decision-making.

In light of rapid and continuous technological advances in food production, it remains crucial to assess both the current and potential future resource and environmental efficiencies of novel technologies. The method to do this is dynamic LCA, also known as prospective or ex ante LCA, which aims to account for changes over time, space and technological performance through integration of technology forecasting methods (van Nielen et al, 2025) (Fig. 2). By way of example, future transformations in energy grids and inorganic nitrogen fertilizer production processes, and on-farm adoption of novel technologies could lead to 30% reductions of the carbon footprint of lettuce production compared to current conditions (Maynard and Quinn 2025). Transitioning from conventional inorganic N fertilizer production to alternative pathways based on renewable energy may, indeed, be a key strategy for sustainable development of the agricultural sector. Continued development and optimization of these alternatives, and the electricity grids underpinning them, are expected to substantially improve environmental performance (Ojelade et al, 2023).

Despite its power and promise, deriving rigorous decision-support information via LCA has long been hindered by data bottlenecks (Hellweg and Milà i Canals, 2014). In dynamic LCA, the burden of data collection is even greater, since more granular data reflecting spatio-temporal and technological changes over time are required (van Nielen et al, 2025). The integration of LCA with recent technological innovations facilitating the collection of “big data”, such as Internet of Things (IoT) and satellite-based sensors, along with techniques for processing these data including machine learning and artificial intelligence (AI), offer solutions (Algren et al, 2021). AI capabilities may overcome data bottlenecks by facilitating efficient data mining, and prediction of data that cannot be collected, such as future inputs and outputs. These techniques may also be used in “surrogate LCA”, whereby prospective models of hypothetical, novel products or systems are developed to support product and system design (Algren et al, 2021). Digital technologies and big data will be key to transforming LCA into a high-resolution, active sustainability diagnosis and management tool. For example, such advances will enable the real-time calculation of food product carbon footprints and support high-leverage mitigation strategies.

Despite its power and promise, deriving rigorous decision-support information via LCA has long been hindered by data bottlenecks.

## Limitations of LCA

Although the utility of LCA for assessing current and future scenarios for sustainable development of the global food system is clear, it is also important to recognize its limitations. LCA may not be well suited to quantify metrics with high uncertainty, such as biodiversity impacts, microplastic pollution or soil health in particular, where clear and aggregable cause-effect relationships cannot be established (Hellweg and Milà i Canals, 2014). Environmental LCA is ultimately capable of addressing only a subset of relevant resource/environmental considerations. Moreover, while parallel techniques for considering a variety of socioeconomic issues (Social LCA and Life Cycle Costing) are available, these remain considerably less developed and infrequently applied compared to environmental LCA (Hellweg and Milà i Canals, 2014).

More importantly, like most science, LCA will never tell us what is the “right” solution when considering technology or management alternatives and the trade-offs among them—whether this is with respect to reducing environmental impacts, improving socioeconomic outcomes such as labor conditions, animal welfare, quality of life, or other goals. Instead, making decisions that support long-term sustainability first requires developing a shared understanding of what those objectives are, and how they interact and are valued relative to each other (Piso et al, 2016). Only once these are clearly defined can the capabilities of LCA be leveraged in evidence-based decision-making for progress towards achieving long-term sustainability goals for the future of food production.

… like most science, LCA will never tell us what is the “right” solution when considering technology or management alternatives and the trade-offs among them…

## Supplementary information

Peer Review File
