# Supplementary material for: Food for thought: The role of life cycle thinking in sustainable food system transitions
Source: EMBO Rep. 2026 Jul 1;27(14):3903–8. doi: 10.1038/s44319-026-00853-x (PMC13400636; doi:10.1038/s44319-026-00853-x)
Supplement: Supplementary file 1 — Peer Review File [file 44319_2026_853_MOESM1_ESM.pdf]

EMBO reports

Food for thought: The role of life cycle thinking in sustainable food system transitions

Nathan Pelletier, Sofia Bahmutsky, Nicole Bamber, Jared Brown, Lauren McNeil, Nicole Sibanda, Shaiyan Siddique, Amandeep Singh, and Ian Turner

Corresponding author(s): Nathan Pelletier (nathan.pelletier@ubc.ca)

Review Timeline:

Submission Date:	16th Jun 26
Accepted:	18th Jun 26

Editor: Holger Breithaupt

Transaction Report: This article was editorially reviewed.
